# Intraoperative dual laparoscopy and neo-rectoscopy for precise excision of bowel endometriosis

**DOI:** 10.1055/a-2440-6251

**Published:** 2024-11-08

**Authors:** Yunxi Zheng, Hao Zhang, Kaikai Chang, Shouxin Gu, Yun Chen, Junjie Xing, Xiaofang Yi

**Affiliations:** 1 92276 Gynecology, Obstetrics and Gynecology Hospital of Fudan University, Shanghai, China; 2 12478 Obstetrics and Gynecology, Shanghai Medical School, Fudan University, Shanghai, China; 3 92276 Radiology, Obstetrics and Gynecology Hospital of Fudan University, Shanghai, China; 4 12520 Colorectal Surgery, Changhai Hospital, Shanghai, China

Surgical treatment for bowel endometriosis poses a significant challenge for gynecologists 1 . The decision between shaving, disc resection, or segmental resection remains uncertain for both gynecologists and general surgeons. More precise surgical intervention reduces tissue damage and lowers recurrence rates 2 . Previously, dual endoscopic techniques have demonstrated significant advantages in detecting esophageal-jejunal anastomotic fistulas 3 . However, there is currently no published evidence of this method being applied to the treatment of endometriosis. Here, we report the use of intraoperative dual laparoscopy and neo-rectoscopy for the precise excision of bowel endometriosis.

A 34-year-old woman presented with progressive dysmenorrhea for 10 years and periodic anal distension for 1 year. Preoperative gynecological examination ( Fig. 1 ) and radiological findings 4 suggested infiltration of the rectal and vaginal walls by endometriotic lesions ( Fig. 2 ). Colonoscopy revealed a 1-cm uneven nodule within the rectal lumen, which was suspected to be an endometriotic lesion ( Fig. 2 ). After obtaining the patient’s consent, laparoscopic surgery was scheduled. Following the shaving of the superficial bowel endometriosis lesions, intraoperative dual laparoscopy and neo-rectoscopy was initiated. Under laparoscopic guidance (Karl Storz 26605BA), a neo-rectoscope was inserted through the anus using a hysteroscopic lens (Olympus A4676A). Dual endoscopy was performed simultaneously by two operators ( Fig. 3 ), allowing for the precise identification of lesion boundaries using an alternating brightness and darkness effect ( Video 1 ).

**Fig. 1:**
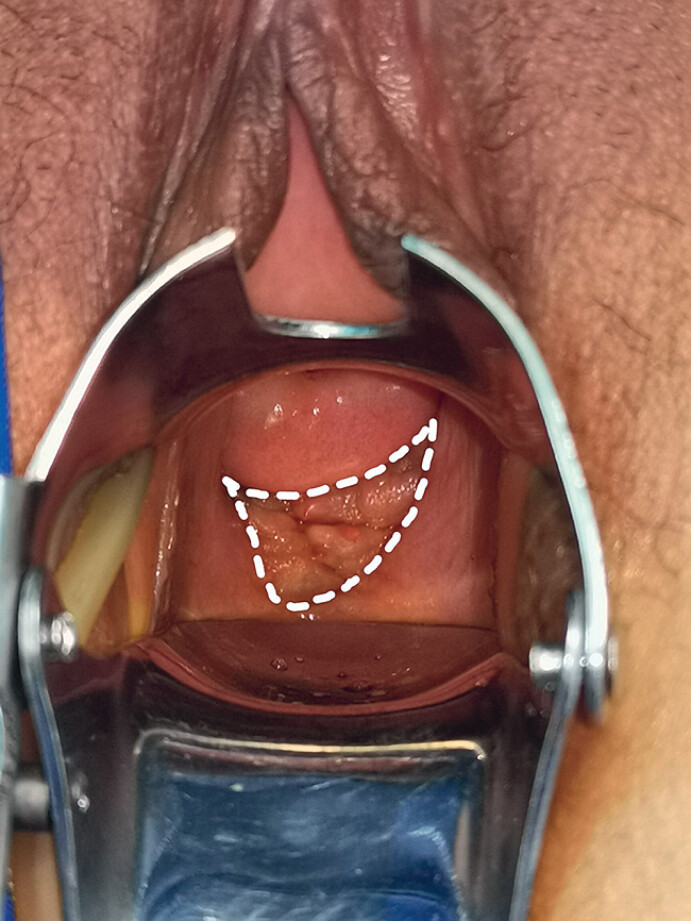
Gynecological examination showing a deep endometriotic lesion involving the vaginal wall (white dashed lines).

**Fig. 2:**
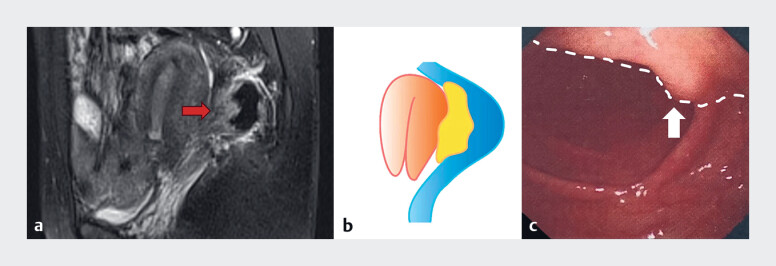
Preoperative evaluation of bowel endometriotic lesions. **a, b** Pelvic MRI findings ( **a** ) and corresponding schematic drawing ( **b** ) of rectovaginal endometriosis, revealing a 2.4 × 1.9-cm solid irregular mass in the pouch of Douglas and local thickening of the anterior rectal wall (red arrow). **c** Colonoscopy confirmed that the bowel endometriotic lesion (white arrow) had infiltrated the full thickness of the rectal wall, compressing almost one-third of the stiff rectal lumen (white dashed line).

**Fig. 3:**
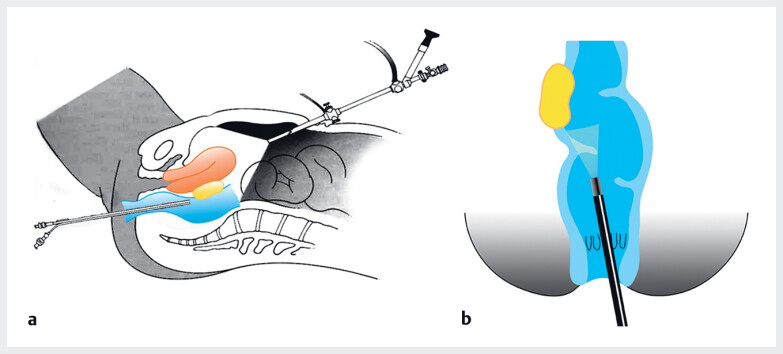
**a** Intraoperative dual laparoscopy and neo-rectoscopy for precise excision of bowel endometriosis. **b** Illustration of neo-rectoscopy during the surgery.

Intraoperative dual endoscopy detection combined with laparoscopy and neo-rectoscopy for precise excision of bowel endometriosis in a 34-year-old woman.Video 1

Once the lesion was fully exposed, a rapid and efficient consultation between the gynecologist and colorectal surgeon ensued. After carefully weighing the risks and benefits of disc excision and segmental resection, a precise rectal disc excision with anastomosis was performed, avoiding the need for a traditional segmental resection of the rectum ( Fig. 4 ). The patient’s bowel function was restored on postoperative day 2. At the 3-month and 4-year follow-up evaluations, her quality of life had significantly improved compared to preoperative assessments, with no signs of impaired bowel function ( Fig. 5 ).

**Fig. 4:**
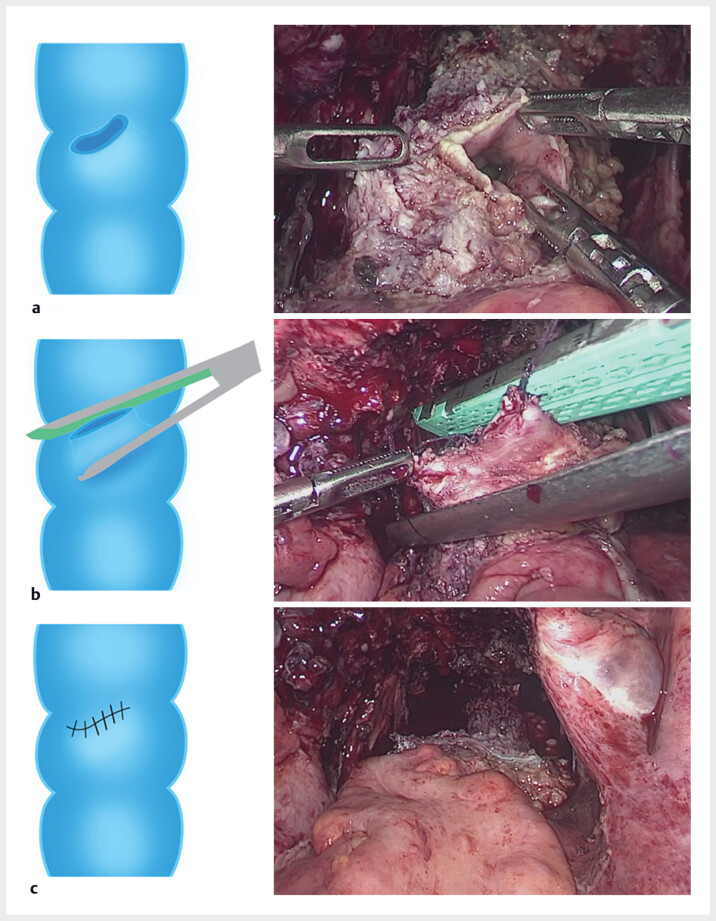
Photographs and illustrations of excision of bowel endometriotic lesion and anastomosis. **a** Precise excision of the bowel endometriotic lesion. **b** Lateral anastomosis using a staple. **c** Post-anastomosis view.

**Fig. 5:**
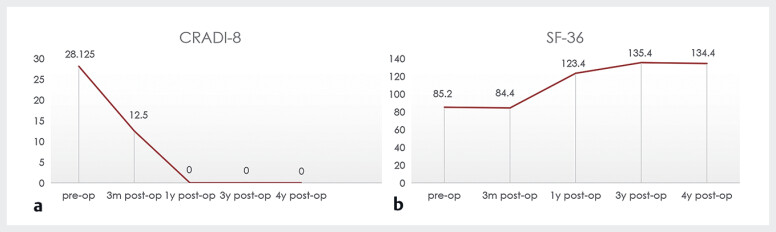
**a, b** Long-term follow-up at 3 months and 4 years revealed significant improvements in bowel function ( **a** ) and quality of life ( **b** ) compared to preoperative evaluation.

Intraoperative dual laparoscopy and neo-rectoscopy can be used to determine the optimal surgical strategy in cases of suspected bowel endometriosis. This technique minimizes tissue damage and recurrence, while also lowering costs. Further clinical studies with larger patient populations and longer follow-up periods are warranted to verify these findings.

Endoscopy_UCTN_Code_TTT_1AT_2AF

## Notes

Endoscopy E-Videos https://eref.thieme.de/e-videos
*E-Videos* is an open access online section of the journal *Endoscopy* , reporting on interesting cases and new techniques in gastroenterological endoscopy. All papers include a high-quality video and are published with a Creative Commons CC-BY license. Endoscopy E-Videos qualify for HINARI discounts and waivers and eligibility is automatically checked during the submission process. We grant 100% waivers to articles whose corresponding authors are based in Group A countries and 50% waivers to those who are based in Group B countries as classified by Research4Life (see: https://www.research4life.org/access/eligibility/ ). This section has its own submission website at https://mc.manuscriptcentral.com/e-videos .

## References

[1] Raimondo D Ianieri MM Raffone A Feasibility of intraoperative proctosigmoidoscopy after discoid bowel resection for deep infiltrating endometriosis: a pilot multicenter study J Minim Invasive Gynecol 2024 31 680 687 10.1016/j.jmig.2024.05.004 38761918

[2] Christiansen A Connelly TM Lincango EP Endometriosis with colonic and rectal involvement: surgical approach and outcomes in 142 patients Langenbecks Arch Surg 2023 408 385 37773225 10.1007/s00423-023-03095-w

[3] Yang Z Bi Y Ren J Dual-endoscopy detection for an esophageal-jejunal anastomotic fistula Endoscopy 2023 55 E868 E869 10.1055/a-2107-2540 37433318 PMC10335861

[4] Zheng Y Gu S Ruan J Bowel wall thickness measured by MRI is useful for early diagnosis of bowel endometriosis Eur Radiol 2023 33 9244 9253 10.1007/s00330-023-09795-7 37498383 PMC10667399

